# Vitamin D Promotes Remyelination by Suppressing c-Myc and Inducing Oligodendrocyte Precursor Cell Differentiation after Traumatic Spinal Cord Injury

**DOI:** 10.7150/ijbs.73673

**Published:** 2022-08-29

**Authors:** Ning Li, Min Yao, Jiaxin Liu, Zhiyuan Zhu, Tsz-Lung Lam, Pingde Zhang, Karrie Mei-Yee Kiang, Gilberto Ka-Kit Leung

**Affiliations:** 1 Department of Surgery, School of Clinical Medicine, LKS Faculty of Medicine, The University of Hong Kong, Queen Mary Hospital, Hong Kong.; 2 Department of Neurosurgery, Zhongda Hospital, Southeast University, Nanjing, China.; 3 School of Pharmaceutical Sciences, Health Science Centre, Shenzhen University, Shenzhen, China.; 4 Department of Functional Neurosurgery, Zhujiang Hospital, Southern Medical University, Guangzhou, China.

**Keywords:** Vitamin D, Myelination, Spinal cord injury, Oligodendrocyte, Differentiation

## Abstract

Demyelination due to oligodendrocytes loss occurs after traumatic spinal cord injury (TSCI). Several studies have suggested the therapeutic potential of vitamin D (VitD) in demyelinating diseases. However, experimental evidence in the context of TSCI is limited, particularly in the presence of prior VitD-deficiency. In the present study, a contusion and a transection TSCI rat model were used, representing mild and severe injury, respectively. Motor recovery was assessed in rats with normal VitD level or with VitD-deficiency after 8 weeks' treatment post-TSCI (Cholecalciferol, 500 IU/kg/day). The impact on myelin integrity was examined by transmission electron microscopy and studied *in vitro* using primary culture of oligodendrocytes. We found that VitD treatment post-TSCI effectively improved hindlimb movement in rats with normal VitD level irrespective of injury severity. However, cord-transected rats with prior deficiency did not seem to benefit from VitD supplementation. Our data further suggested that having sufficient VitD was essential for persevering myelin integrity after injury. VitD rescued oligodendrocytes from apoptotic cell death *in vitro* and enhanced their myelinating ability towards dorsal root axons. Enhanced myelination was mediated by increased oligodendrocyte precursor cells (OPCs) differentiation into oligodendrocytes in concert with c-Myc downregulation and suppressed OPCs proliferation. Our study provides novel insights into the functioning of VitD as a regulator of OPCs differentiation as well as strong preclinical evidence supporting future clinical testing of VitD for TSCI.

## Introduction

Traumatic spinal cord injury (TSCI) is a devastating neurological condition that causes disturbances to sensory, motor, and autonomic functions 1. The management of TSCI patients currently relies on surgical decompression in the acute phase, followed by anti-inflammatory and analgesic medications for symptomatic relief. In the chronic phase, intensive physiotherapy and rehabilitation is often required for years 2. Demyelination and deficient remyelination are major pathological events that contribute to poor functional recovery following TSCI 3, 4. Effective treatments targeting the neurological system are very limited.

Oligodendrocytes (OLs) are specialized glial cells in the central nervous system (CNS) responsible for the formation and maintenance of myelin sheath. It has been reported that focal death of OLs can lead to extensive demyelination, axonal shrinkage, and even neuronal damage, since each OL can support tens of distinct axons 5, 6. Poor recovery following TSCI demyelination may be largely due to the limited self-renewal capacity of differentiated OLs 7. Oligodendrocyte precursor cells (OPCs) are the progenitors of OLs. Previous studies have reported promising results on stem cell therapy for acute spinal cord injury, including OPCs transplantation, to promote myelination 8, 9. Nevertheless, the rescuing effects are often compromised by the hostile microenvironment of the injury site, resulting in inadequate remyelination and functional recovery 10. Therapeutic agents that promote OL-induced remyelination is a promising target for neurotrauma research.

Vitamin D (VitD) is increasingly recognized as a hormone with important biological functions and therapeutic potentials for CNS conditions such as ischemic stroke and traumatic brain injury 11-13. VitD alone has been found to promote neurological recovery after TSCI in animals, mainly through its immune-modulatory and anti-apoptotic effects 14, 15. Thus, the association between VitD status and neurological performance after TSCI is receiving increasing attention. Moreover, among chronic spinal cord injury patients, those with VitD deficiency have been shown to have worse neurological outcomes than patients with normal VitD levels 16, 17. VitD status has been suggested to be an independent predictor of physical performance after TSCI 18. The neuroprotective function of VitD has also been established in multiple sclerosis, a demyelinating disease of the CNS 19. However, the therapeutic potential of VitD treatment for motor function recovery in the context of VitD deficiency remains unclear.

This study investigated the therapeutic effects of VitD treatment post-TSCI, with a particular focus on examining how pre-TSCI VitD status may affect motor recovery. We also aimed to explore the molecular mechanism underlying the effect of VitD on OLs.

## Methods

### Animals and study design

A total of 60 male Sprague Dawley (SD) rats weighing 70-90g, and 18 male C57/6N mice weighing 25-30g and postnatal (P1) SD rats were obtained from the Laboratory Animal Unit of our institution. All operations were performed according to guidelines approved by the Committee on the Use of Live Animals for Teaching and Research (CULATR). Animals were housed in standard conditions and given ad libitum access to normal or VitD deficient rodent chow and water.

**Figure 1:**
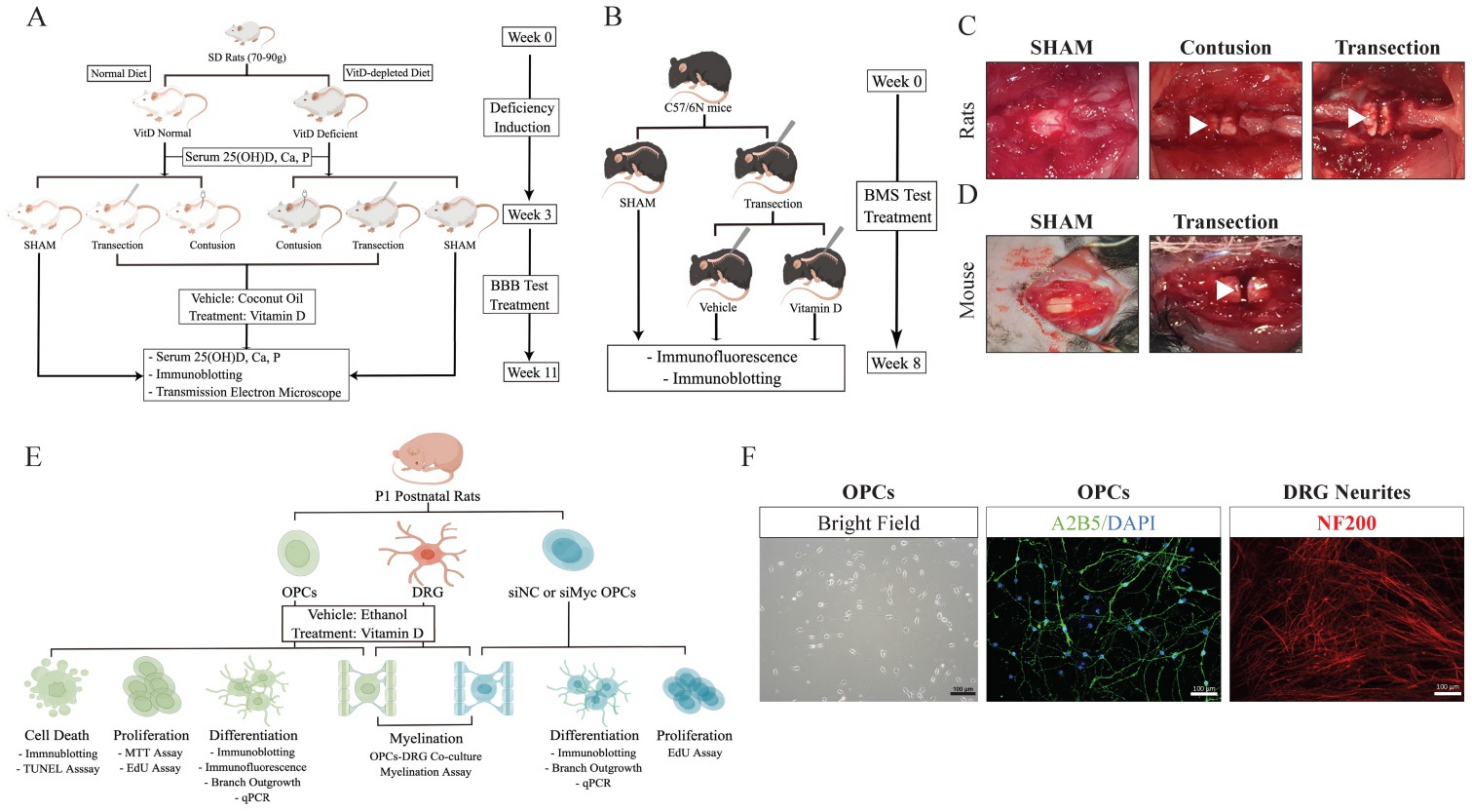
** Graphical illustrations for study designs. (A and B)** *In vivo* studies for rats and mice. **(C and D)** Operative microscopic demonstrations for rat and mice models are shown. White arrow heads indicate the injury sites. **(E)** Design for *in vitro* study. **(F)** Demonstration of OPCs and DRT neurites. OPCs were identified using A2B5 (green) and DAPI (blue). Neurites were identified with NF200 (red).

For the *in vivo* study, two cohorts of SD rats, VitD-normal and -deficient, were enrolled. Two TSCI models, contusion and transection, were induced to create gradient severities. Thus, animals were randomized into 5 groups in each cohort: SHAM, Contusion-VitD, Contusion-Vehicle, and Transection-VitD, and Transection-Vehicle (n=6 each). Cholecalciferol VitD (500 IU/kg/day, Ddrops) was orally administered 2h after injury, and then given q.d. for 8w. Coconut oil was used as vehicle drug. Hindlimb motor function was recorded on the second day and every week afterwards. Serum 25(OH)D, calcium, and phosphorous levels were examined before injury and at the endpoint of the study. Tissues were harvested for immunoblotting, transmission electron microscopic (TEM) experiments (Figure 1A,C). A total of 3 groups of VitD-normal mice (n=6 each) subject to transection TSCI or SHAM were included to further confirm the therapeutic effects of VitD (Figure 1B,D).

For the *in vitro* experiment, primary OPCs and dorsal root ganglion (DRG) cells were raised from P1 postnatal SD rats. The effects of 1,25(OH)2D3 (Calcitriol, 100 nM, Sigma-Aldrich) on OPCs proliferation were tested using Thiazolyl Blue Tetrazolium Bromide (MTT) and 5-Ethynyl-2'-deoxyuridine (EdU) proliferation assays. VitD's impacts on OPC maturation and myelin formation capabilities were assessed using different platforms including immunoblotting, quantitative polymerase chain reaction (qPCR), and *in vitro* morphological and functional assays. Molecular mechanisms were further explored using RNA interference technology (Fig. 1E,F).

### Induction of VitD-deficient rats

VitD deficiency was induced in rats according to literature 20. They were fed with a VitD-depleted diet (No VitD, 0.47% calcium, 0.3% phosphorus, TD.89123, Harlan Tekled) for 3w. VitD-normal rats were housed in the same condition, but fed with normal rodent diet (2.3 IU/g VitD, 0.81% calcium, 0.64% phosphorus, 5053, Picolab) in parallel. Hypovitaminosis D was confirmed by ELISA of serum 25(OH)D level 1d before TSCI model.

### Traumatic spinal cord injury models

Animals were anesthetized by intraperitoneal injection of Xylazine (10 mg/kg) and Ketamine (80 mg/kg). A midline longitudinal incision was made and the region of interest (T9 - T11 levels of the thoracic spine) was exposed. A laminectomy was performed at T10 under the Zeiss microscope. The spinal cord was left uninjured and the dura kept intact in SHAM animals. In the experimental groups, transection injury was introduced by a transverse cut from dorsal surface to ventral floor using a sharp surgical blade. The model was considered a success when an obvious lesion cavity was witnessed. Contusion injury was introduced by acute compression with a 10g vessel clip (Kent Scientific) for 5 min. A hindlimb convulsion followed by full paralysis indicated the successful establishment of injury.

### Assessment of serum 25-hydroxy-vitamin D, calcium and phosphate levels

Blood samples were collected from the lateral tail veins and allowed to clot at room temperature protected from light, followed by centrifugation at 1,500*g* for 10 min at 4°C. Supernatant serums were collected. 25(OH)D level was detected using 25(OH)D ELISA kit (Immunodiagnostic Systems); calcium and phosphate levels were detected using calcium assay kit and phosphate assay kit (both Abcam) according to the manufacturer's instructions.

### Motor function assessments

The Basso, Beattie, and Bresnahan (BBB) Locomotor Rating Scale 21 and the Basso Mouse Scale (BMS) for Locomotion 22 were used to evaluate hindlimb motor recovery in rats and mice, respectively. Scores were recorded at the following time points: before the operation, 1d after injury, and weekly afterwards. Scores were evaluated by two individuals, the average score from the two observers was used.

### Immunoblotting

Three animals from each group were randomly selected, anesthetized and transcardially perfused with pre-chilled saline. A 4 cm segment of spinal cord including the injury site was quickly harvested and divided into three 1 cm segments on ice: 0.5cm rostral and caudal from the injury site, and the epicenter. Proteins were homogenized and extracted using RIPA buffer (Cell Signaling) with protease inhibitor cocktail (Roche). Protein lysates were electrophoresed on SDS polyacrylamide gel and transferred to PVDF membranes. After blocking, membranes were incubated with myelin basic protein (MBP, Millipore for cell and Cell Signaling for tissue), cleaved poly (ADP-ribose) polymerase (cleaved PARP), total Caspase 3, cleaved caspase 3, c-Myc, Actin and GAPDH (all from Cell Signaling) at 4 °C overnight. After rinsing, HRP-conjugated anti-rabbit or anti-mouse secondary antibodies (both from Cell Signaling) were incubated for 2h. Chemiluminescent reagents were added, and signals were visualized using X-ray films.

### Transmission electron microscopy on spinal cord specimens

Tissues for TEM were prepared according to our published protocols 23. Briefly, 3 animals per group were anesthetized and transcardially perfused with pre-chilled saline followed by 4% paraformaldehyde solution (PFA) 8w after injury. Spinal cord specimens were harvested, and post-fixed in 2.5% glutaraldehyde in 0.1 M phosphate buffer and then in 1% osmium tetroxide overnight. After progressive dehydration in in gradient ethanol (30% to 100%), tissues were further processed in propylene oxide and Epon resin. Ultrathin section was cut in 90nm using copper grids and stained with 3% uranyl acetate and 1% lead citrate. Three tissue sections were randomly selected from each sample, and six non-overlapping fields were picked in the white matter from each slice using Philips CM100 transmission electron microscope. Axons with a diameter ranging from 800 to 1000 nm were opted for myelin measurement. G-ratio (the ratio between the inner and the outer diameter) was further analyzed using ImageJ software 24. Images were demonstrated in 5,200X and 28,500X magnification except for the epicenter in transection group due to extensive demyelination, where lower power at 1,650X and 5,200X were used instead.

#### Fetal bovine serum charcoal stripping

To remove residual VitD in the culture medium, fetal bovine serum (FBS, Thermo Scientific) was subjected to stripping according to literature 25. Briefly, FBS was incubated with 2.5% activated charcoal powder (Sigma-Aldrich) and incubate at 4 °C overnight with gentle shaking. FBS was then centrifuged at 2000*g* at 4 °C for 15 min. Supernatant (C-FBS) was collected, filtered through a 0.22μm syringe filter and stored at -20 °C.

### Primary culture of rat oligodendrocyte precursor cells

Primary culture of OPCs was prepared according to literature 26. Postnatal rats (P1) were decapitated after euthanasia procedure with CO_2_. Brain cortex isolated from postnatal rat was cut into 1mm^3^ pieces in cold Hank's Balanced Salt Solution (HBSS, Thermo Scientific), and digested in DNase I and trypsin for 15 min at 37 °C. Mixed primary cells were cultured with DMEM20S (DMEM medium supplemented with 4 mM L-glutamine, 1 mM sodium pyruvate, 10% C-FBS, 50 U/ml penicillin and 50 µg/ml streptomycin, from Sigma-Aldrich and Thermo Scientific) in poly-D-lysine-coated flasks (Sigma-Aldrich) for 10d. Microglial cells were removed by shaking on a horizontal orbital shaker at 200 rpm 37 °C overnight. On the second day, supernatant was collected and cultured with ultra-low culture dish (Corning) to further remove contaminated microglia and fibroblasts. Finally, OPCs were ready for *in vitro* experiments after 5-6 d in the proliferation medium (Neurobasal medium supplemented with 1:50 B27 Supplement, 1:100 GlutaMax, 10 ng/ml bFGF and 10 ng/ml PDGFα, from Thermo Scientific and PeproTech), or for differentiation in differentiation medium (proliferation medium without bFGF and PDGFα).

### Primary culture of dorsal root ganglia

DRG were collected and cultured according to our published work 27. Spinal columns were harvested from P1 rats, and cords were removed to expose foramen intervertebral. Ganglia were digested in collagenase I (Sigma-Aldrich) in DMEM at 37 °C for 60 min, followed by trypsin for another half an hour. DRG were purified in DMEM containing 15% bovine serum albumin (BSA) to remove fibroblast. Supernatant were collected, and DRG cells were seeded onto poly-D-lysine-coated (Sigma-Aldrich) coverslips in 4-well dishes in Neurobasal medium supplemented with 1:50 B27 Supplement, 1:100 GlutaMax and 50 ng/mL nerve growth factor (NGF, Sigma-Aldrich). Proliferating contaminative cells such as fibroblasts were removed by 10 μM cytosine arabinoside (Ara-C, Sigma-Aldrich) every 4d. After 9-10d of culture, DRG were ready for *in vitro* experiments.

### *In vitro* myelination assay in OPCs-DRG co-culture system

According to our previous work 27, OPCs were seeded onto DRG-plated coverslips at a OPCs:DRG ratio of 1.5×10^4^ : 5×10^4^ cells per well with supplementation of NGF. After full attachment, bFGF and PDGFα were withdrawn to induce OPCs differentiation for 6d. Cells were then fixed for MBP and NF200 immunofluorescence staining.

### Immunofluorescence staining

For *in vivo* study, mice spinal cords at T10 were collected for cryosections. After air dry and blocking with 10% normal goat serum, sections were immersed in pre-heated 0.01 M, PH 6 citrate buffer and 3% hydrogen peroxide for antigen retrieval. For *in vitro* staining, cells were fixed with -20 °C pure methanol, followed by blocking with PBS-T blocking buffer. Primary antibodies including MBP (Cell Signaling) and NF200 (Abcam) were incubated overnight, followed by secondary antibodies on the second day. Slides were mounted, and images were acquired using Carl Zeiss LSM 800 confocal microscope. Six randomly selected non-overlapping fields from each section were analyzed.

### *In situ* cell death detection (TUNEL Assay)

Differentiated OLs were fixed with 4% PFA and then permeabilized with 0.1% Triton X-100 in 0.1% sodium citrate in PB buffer. Cells were incubated in terminal deoxynucleotidyl transferase dUTP nick end labeling (TUNEL) reaction mixture (Roche) for 1h in 37 °C humidified atmosphere, and then mounted with Fluoromount-G (Thermo Fisher). Tests were performed in triplicates, and DAPI (blue) and TUNEL (green) positive cells were counted in six randomly selected non-overlapping fields in each slide for statistical analysis.

### Cell proliferation assays

Cell proliferation was assessed using Thiazolyl Blue Tetrazolium Bromide (MTT, Sigma-Aldrich) assay and 5-Ethynyl-2'-deoxyuridine (Edu, Ribobio) assay. For MTT, OPCs were seeded into poly-D-lysine-ornithine coated 96-well plates at the density of 1×10^4^ cells/well. After six days, MTT solution was added and incubated for 3h at 37 °C. DMSO was added into each well for 15min until full dissolution. Absorbance was measured at OD at 590nm. Samples were tested in triplicate. For Edu assay, reagent was added into medium, and incubated at 37 °C for 2h. Cells were then fixed, neutralized with glycine, and permeabilized with 0.5% TritonX-100 in PBS. Subsequently, cells were stained with Apollo solution and further counter-stained with Hoechst33342. Six non-overlapping fields were randomly selected for cell counting and imaging.

### Transient knockdown of *Myc* in OPCs by small-interfering RNA

*Myc-*specific trilencer-27 siRNA oligo duplexes, and a trilencer-27 universal scrambled negative control siRNA duplex were obtained from Origene Technologies. 50 nM siRNA were transfected into OPCs using Lipofectamine RNAiMAX regent (Thermo Scientific) according to the manufacturer's instructions. Cells transfected with siRNA for 3d were harvested for further experiment.

Myc siRNA duplex sequence: 5' AGGACUUACUGAGGAAACGGCGAGA 3'.

### Quantitative polymerase chain reaction

RNA was extracted from tissue or cells using Trizol reagent. RT-PCR was conducted with genomic DNA elimination followed by reverse transcription into cDNA using PrimeScript RT reagent kit (Takara). qPCR was performed in a 20 μL SYBR Green reaction mixture. *Gapdh* was used as the internal control. qPCR reaction was performed using ViiA-7 Real-Time PCR system. mRNA expressions were analyzed using 2-∆∆CT calculation method after normalization with *Gapdh*. Primer sequences are as follows:

- Gapdh Forward: 5' GACATGCCGCCTGGAGAAAC 3';
- Reverse: 5' AGCCCAGGATGCCCTTTAGT 3'.
- Myc Forward: 5' TTCCACGGCCTTCTCTTCTT 3';
- Reverse: 5' GGGTTGCCTCTTTTCCACAG 3'.
- Mbp Forward 5' ATGGCATCACAGAAGAGACCCTCA 3';
- Reverse: 5' TAAAGAAGCGCCCGATGGAGTCAA 3'.

### Statistical Analysis

GraphPad Prism 9 was used for statistical analysis. Results were presented as mean ± standard deviation (SD) or standard error of mean (SEM) as indicated. T-test was used to analyze the difference between two groups. One-way analysis of variance (ANOVA) with Tukey's multiple comparison test was used to analyze the differences between groups. *P* value less than 0.05 was considered to be statistically significant.

## Results

### Vitamin D treatment promotes motor recovery after traumatic spinal cord injury in rats and mice

**Figure 2:**
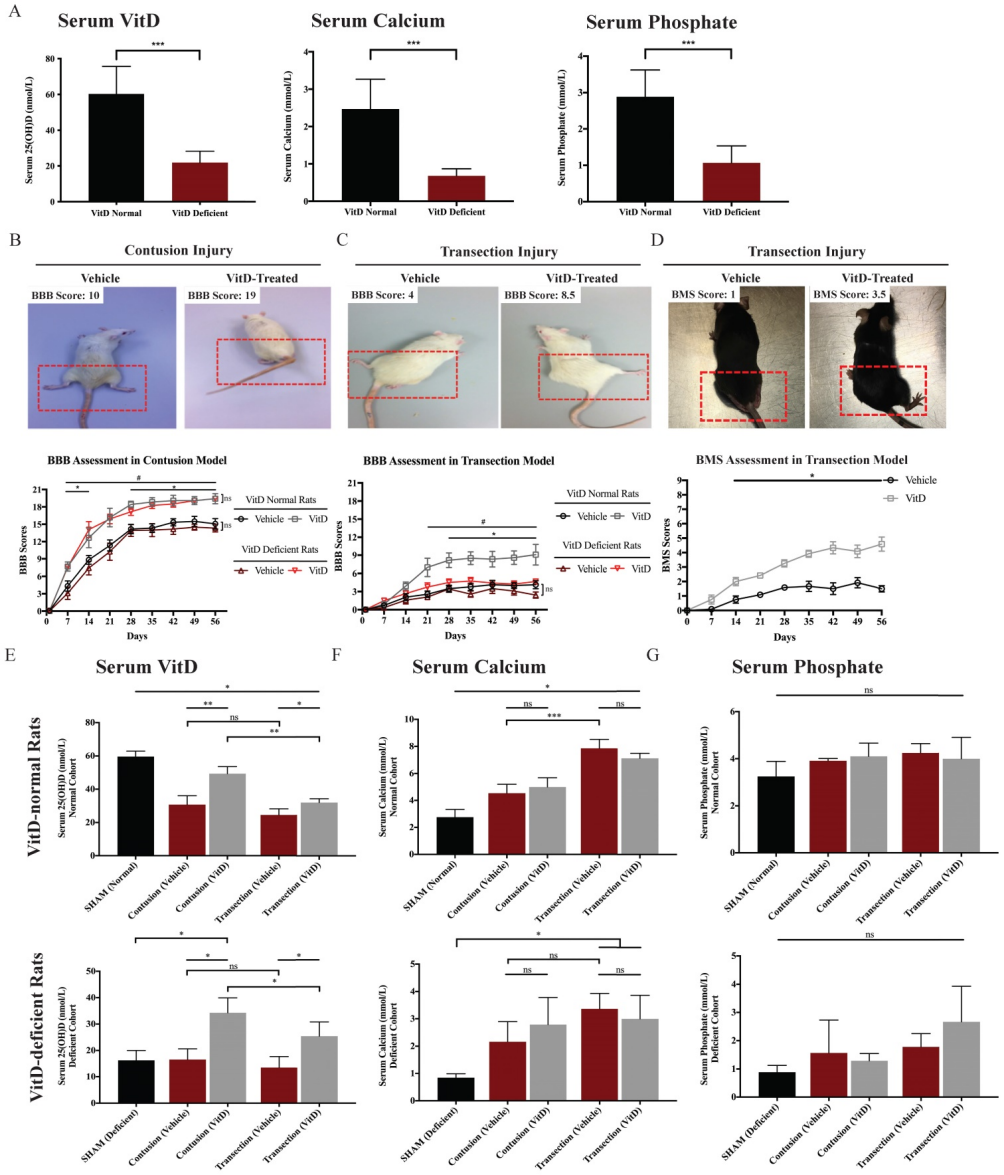
** VitD treatment promoted motor recovery in rats and mice after TSCI. (A)** VitD deficiency was confirmed by serum 25(OH)D ELISA test. Concurrent decreases of serum calcium and phosphate were seen (n=12). **(B)** BBB result in contusion cohort is shown with two representative photos illustrating BBB 10 and 19. (^*^p < .05 between vehicle- and VitD-treated rats in the normal cohort, ^#^*p* < .05 between treated and untreated animals in the VitD-deficient cohort, n=6). **(C)** BBB result in transection cohort is shown, together with two representatives for BBB 4 and 8.5 (^#^*p* < .05 between vehicle- and VitD-treated rats in the normal cohort, ^*^p < .05 between treated normal and deficient rats). **(D)** BMS record demonstrates that VitD treatment enhanced motor function recovery from Day 14 onward (^*^p < .05, n=6). Animal photos reveals more significant postural deficits in the vehicle mouse in comparison with the VitD-treated mouse. **(E-G)** Serum 25(OH)D, calcium and phosphate fluctuation by the end of 8w are shown. VitD treatment increased serum 25(OH)D but resulted in no changes in calcium or phosphate levels (n=3). Data were presented as mean ± SEM for behavioral tests, and mean ± SD for the rest. *, ** and *** represent p < .05, .01 and .001.

VitD deficiency was induced through a modified diet for three weeks and confirmed by ELISA. The mean serum 25(OH)D level of VitD-deficient rats was 21.84 ± 2.03 nmol/L, compared to 60.27 ± 4.44 nmol/L in VitD-normal rats. Concurrent reductions in serum calcium and phosphate were detected in VitD-depleted rats (Figure 2A). TSCI was induced either through contusion or transection injury, representing different severity levels. Although the contusion model was characterized by relatively mild spinal cord injury, complete paraplegia, which is indicative of a successful TSCI model, was common 1 or 2d after inducement due to spinal shock.

As expected, the contusion and transection injury cohorts showed an obvious difference in the rate of motor recovery, reflecting clinical TSCI cases with different injury severity levels (Figure 2B and 1C). After contusion injury, VitD treatment increased the motor recovery rates from Day 7 onward in both the VitD-deficient and normal groups (^*^p < .05 and ^#^*p* < .05; Figure 2B). In the transection animals, while VitD started to show therapeutic benefits in normal rats 3w after injury (^#^*p* < .05, Figure 2C), but was ineffective in the VitD-deficient cohort. When examining the impacts of pre-hypovitaminosis on functional recovery, no statistical difference was reached between VitD-normal and -deficient rats after cord contusion either with or without VitD treatment. In contrast, a better response to treatment was observed in VitD-normal rats after transection injury, as they showed a better movement since week 4 when compared to prior-deficient ones (^*^p < .05, Figure 2C). In the parallel study using transection model in mice, VitD-treated animals showed better hindlimb movement from week 2 onward (Figure 2D). It is worth noting that untreated mice generally had a worse general condition such as weight loss and slower bladder function recovery (data not shown). This suggests that VitD may not only enhance neuronal recovery but may also confer beneficial effects on general health after severe TSCI. The overall findings are suggestive of the potential therapeutic benefits of VitD supplementation in animals with normal pre-traumatic VitD level irrespective of the severity of injury, and in animals with prior VitD-deficiency suffering from relatively mild injury. VitD supplementation does not appear to help recovery in animals prior VitD-deficiency and severe injury.

Serum 25(OH)D level dropped by more than half in VitD-normal rats 8w after TSCI. Conversely, the injury did not further reduce serum VitD level in VitD-deficient rats (Figure 2E). Serum calcium levels increased after TSCI, especially after transection injury, which resulted in more severe hypercalcemia, possibly due to increased bone resorption caused by immobilization. However, this phenomenon was not observed in VitD-deficient rats (Figure 2F). Blood phosphate levels did not change after TSCI or VitD treatment in all groups (Figure 2G). Notably, VitD at the experimental dosage did not induce additional hypercalcemia or hyperphosphatemia.

### VitD treatment preserved myelin sheath integrity

**Figure 3:**
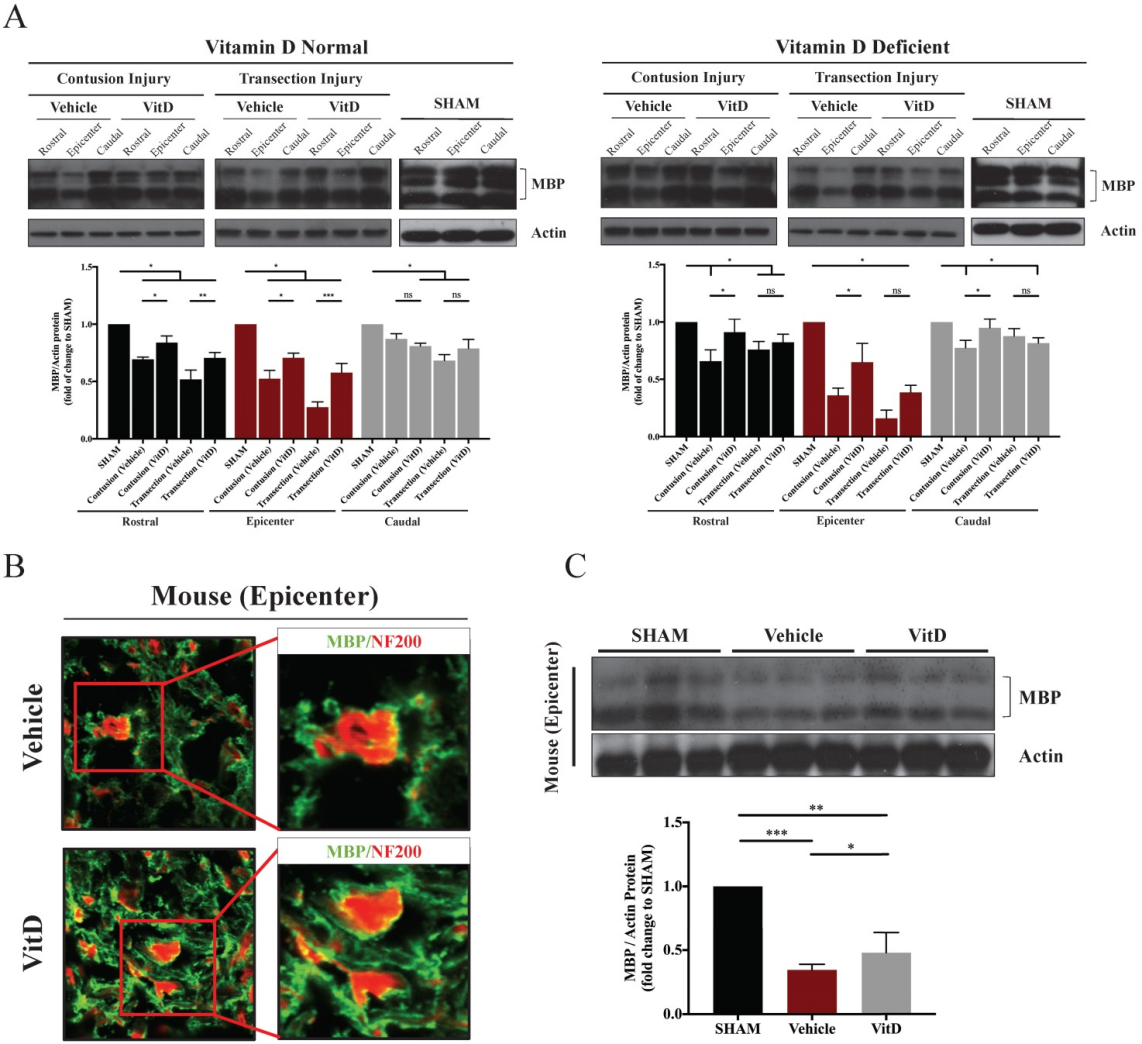
** MBP levels in different groups showing the preservative benefit of VitD on myelin protein. (A)** MBP levels at the epicenter, rostral and caudal sections were analyzed. Western blotting showed significant decreases in MBP levels after contusion and transection injury in the epicenters, and VitD treatment ameliorated such damage except for the deficient animals with transection injuries. Rostral rather than caudal spinal cords also suffered from obvious myelin damages in both cohorts. **(B)** Immunofluorescence staining in the epicenter of mouse spinal cords with transection injury showed increased myelination (indicated by MBP, green) surrounding the axons (indicated by NF200, red). Original magnification 200X (left) and 400X (right). **(F)** Western blotting of MBP protein expression in the epicenter also showed an obvious increase in MBP after VitD treatment. Data are presented as mean ± SD, and n=3.^ *^p < .05, ^**^p < .01, ^***^p < .001.

Demyelination indicated by the decrease of MBP expression was seen in TSCI specimens. The extent of myelin damage correlated with the severity of injury: MBP expression was considerably lower in transection injury tissues than in contusion injury tissues (Figure 3A). Surprisingly, demyelination was also observed in both the rostral and caudal segments 0.5 cm away from the injury site, and was more severe in an antidromic direction (i.e., in the rostral end compared to the caudal end).

In line with BBB score results, VitD treatment was more effective in the contusion injury cohort, as it preserved MBP regardless of pre-injury VitD status, not only at the injury site but also in the rostral segment (VitD treatment compared to vehicle controls in the epicenter and rostral segment in both VitD-normal and VitD-deficient rats: *p < .05). In contrary, VitD treatment was less effective in complete injury following transection cut, particularly in the VitD-deficient cohort; the increase in MBP was not seen after treatment (Figure 3A). The results were consistent in mice with transection injury. In the vehicle group, more than half of the axonal surface stained with NF200 was not surrounded by MBP-stained myelin, suggesting substantial myelin loss. In contrast, the axonal networks were well preserved in VitD-treated mice (Figure 3B). MBP expression was also significantly increased after VitD treatment (Figure 3C).

**Figure 4:**
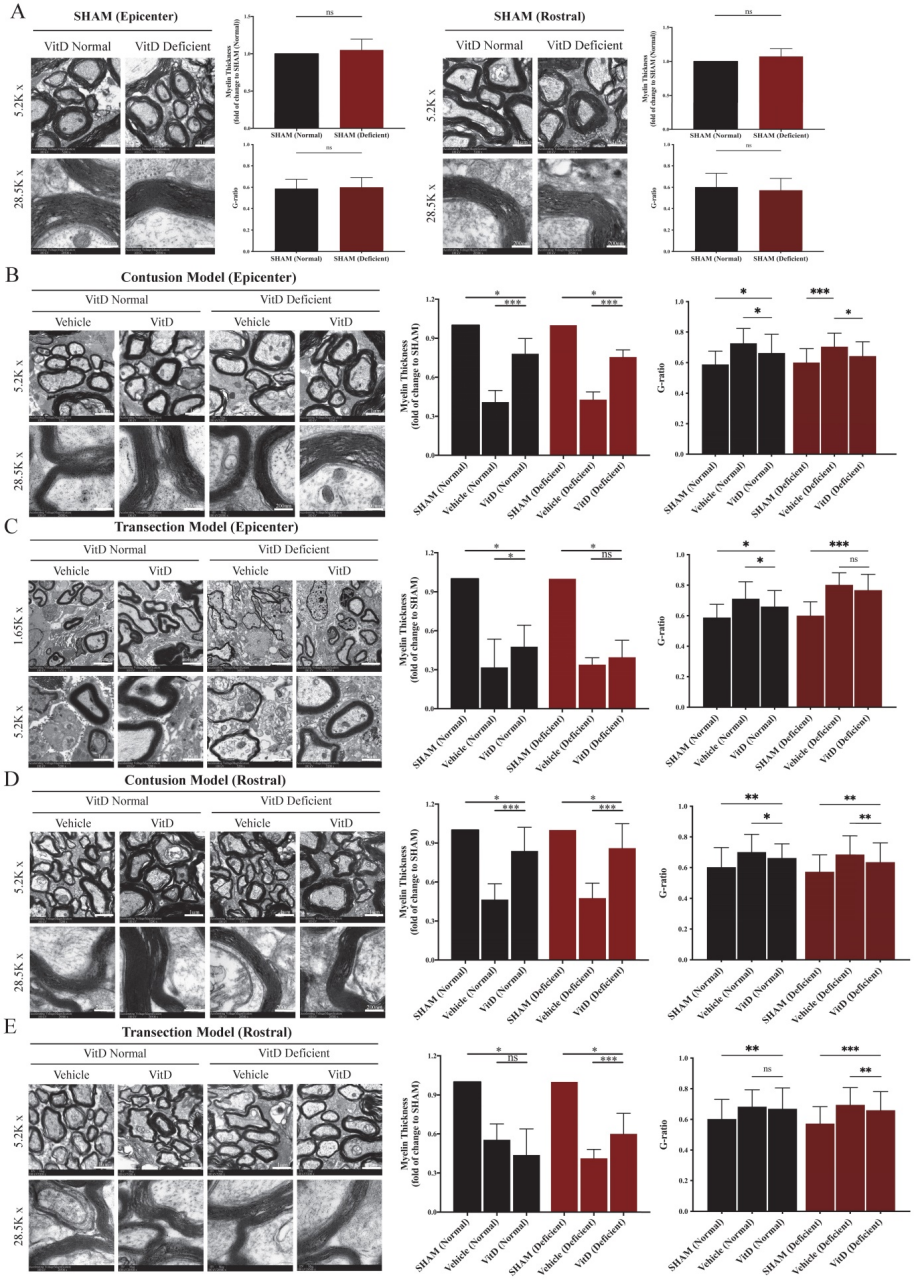
** TEM demonstrated that VitD treatment preserved myelin sheath integrity.** Myelin integrity in the epicenters and rostral segments were evaluated by myelin thickness measurement and g-ratio analysis. **(A)** VitD deficiency did not cause myelin damage in SHAM animals. **(B and C)** Both contusion and transection injury caused significant myelin breaks in the epicenters. In line with the functional and MBP analysis, VitD increased the integrity of myelin in all pairs except for the transection rats with prior VitD-deficiency. **(D and E)** Rostral tissues showed similar trend that demyelination could be clearly witnessed in both models, and VitD also showed its therapeutic values in all contusion groups. Again, it became less effective in rats with transection injury. Data are shown as mean ± SD, and n=3. Scale bars are 4µm, 1µm and 200nm in 1.65K, 5.2K and 28.5K magnified images, respectively. ^*^p < .05, ^**^p < .01, ^***^p < .001.

The integrity of myelin sheath was examined using TEM and evaluated by myelin thickness and g-ratio. High consistency was seen between the two methods. VitD-deficiency did not induce abnormality on myelin sheath integrity in the SHAM groups (Figure 4A). Significant degeneration was observed after contusion and transection injury in both the epicenter and the rostral segment (Figure 4B-E). Transection injury introduced a more severe insult to the myelin sheath, as evidenced by reduced myelin sheath thickness, a higher g-ratio, and a larger number of naked axons (Figure 4C). VitD treatment was able to protect myelin tissue in both normal and VitD-deficient animals with contusion injury at the epicenter as well as the rostral segment (Figure 4B,D). As reflected in functional assessment, transected rats were less likely to benefit from VitD treatment from the perspective of myelin integrity (Figure 4C).

### VitD treatment protected OLs from apoptotic cell death and induced OPCs differentiation

**Figure 5:**
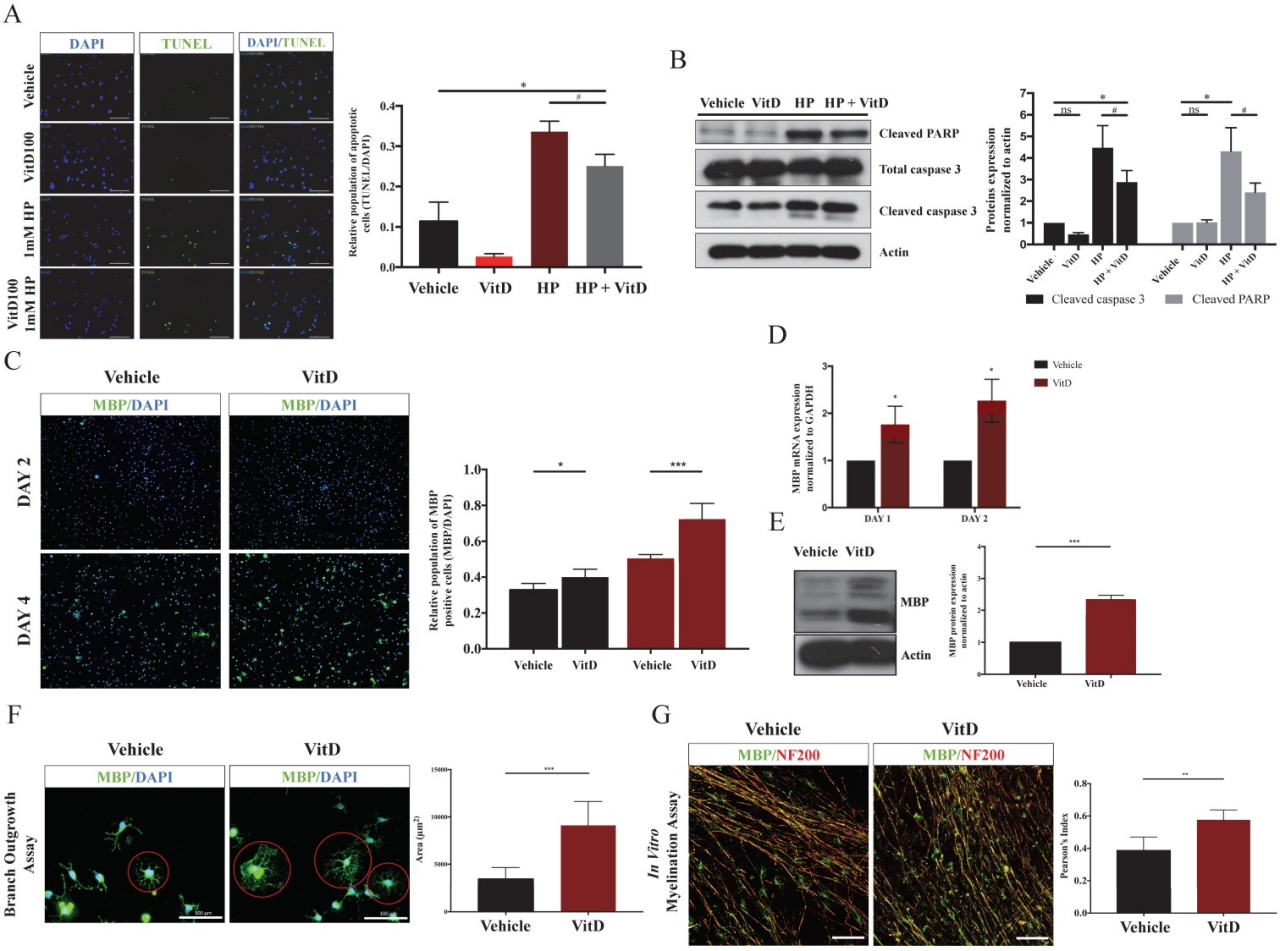
** VitD protected OLs from apoptosis and facilitated OPCs differentiation and myelination* in vitro*. (A)** A pro-survival effect of VitD was demonstrated by TUNEL assay in OLs exposed to HP. Green fluorescence indicated TUNEL-positive apoptotic cells. Original magnification 100X. **(B)** Mechanistic analysis showed that VitD suppressed the cleavage of PARP and caspase 3, two important apoptotic markers. **(C)** MBP (green) and DAPI (blue) immunofluorescence staining in an OPCs culture after VitD treatment (100 nM) for 2 and 4 days. The ratio of MBP-positive cells to the total number of DAPI cells increased significantly in VitD-treated OPCs. Original magnification 100X. **(D)** qPCR and **(E)** Western blotting also showed increased MBP mRNA and protein expression after VitD treatment in OPCs. **(F)** A branch outgrowth assay of OPCs demonstrated the enhanced outgrowth ability or the extent of oligo-maturation after 5d of VitD treatment. The outgrowth area was measured as a representative index (Scale bar = 100µm). **(G)** MBP (green) and axonal marker NF200 (red) immunofluorescence staining in a OPCs-DRG coculture after 6d of VitD treatment. Pearson's index was used to analyze overlapping staining as a sign of axonal myelination (Scale bar = 100µm). Data are shown as mean ± SD.^ *^p < .05, ^**^p < .01, ^***^p < .001.

To examine whether VitD could protect OLs from exogenous insults, hydrogen peroxide (HP) was used to induce OLs apoptosis and mimic the apoptotic signal induced in TSCI *in vitro*. VitD treatment rescued OLs from HP-induced apoptosis (^#^*p* < .05 between HP and HP + VitD; Figure 5A) by suppressing cleaved PARP and cleaved caspase 3 (Figure 5B). Furthermore, VitD treatment promoted differentiation of OPCs into OLs after 2d and 4d (Figure 5C), as concurrently indicated by elevated MBP mRNA (Figure 5D) and protein (Figure 5E) expression. A branch outgrowth assay was performed to assess the extent of oligo-maturation, a morphological characteristic of functional OLs. VitD-treated OLs showed more neurites with further outreach, and the inter-process networks were more robust compared to the controls (Figure 5F). The myelinating ability of OLs was assessed in a DRG-OPCs coculture system after VitD treatment for 6d. Pearson's index (merged MBP and NF200 signals) increased 1.5-fold in the VitD-treated system, suggesting that myelination of DRG axons were facilitated by VitD treatment (Figure 5G).

### VitD promotes oligodendrocyte differentiation by suppressing c-Myc signaling

**Figure 6:**
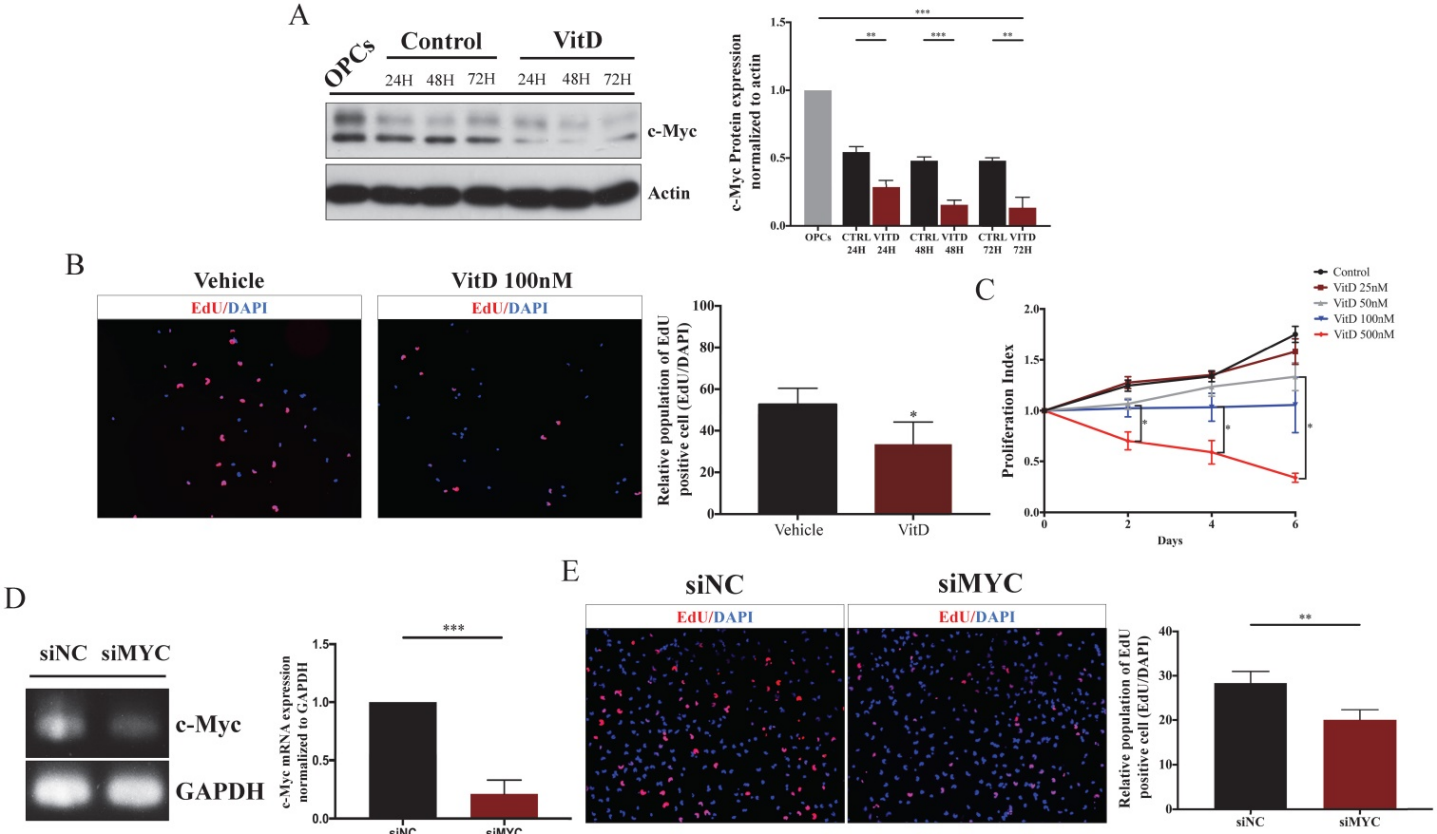
** VitD decelerated OPCs proliferation by c-Myc suppression. (A)** Western blotting showed c-Myc was suppressed by VitD during OPCs differentiation in 72h. **(B)** EdU proliferation assay (EdU: red and DAPI: blue) showed a smaller amount of proliferating OPCs upon 100nM VitD treaetment. Original magnification 100X. **(C)** MTT assay demonstrated that VitD inhibited the proliferation of OPCs in a dose dependent manner. **(D)** qPCR confirmed siRNA-mediated c-Myc knockdown in OPCs. **(E)** EdU proliferation assay showed a reduced proliferation in siMyc OPCs. Original magnification 100X. Data are shown as mean ± SD.^ *^p < .05, ^**^p < .01, ^***^p < .001.

**Figure 7:**
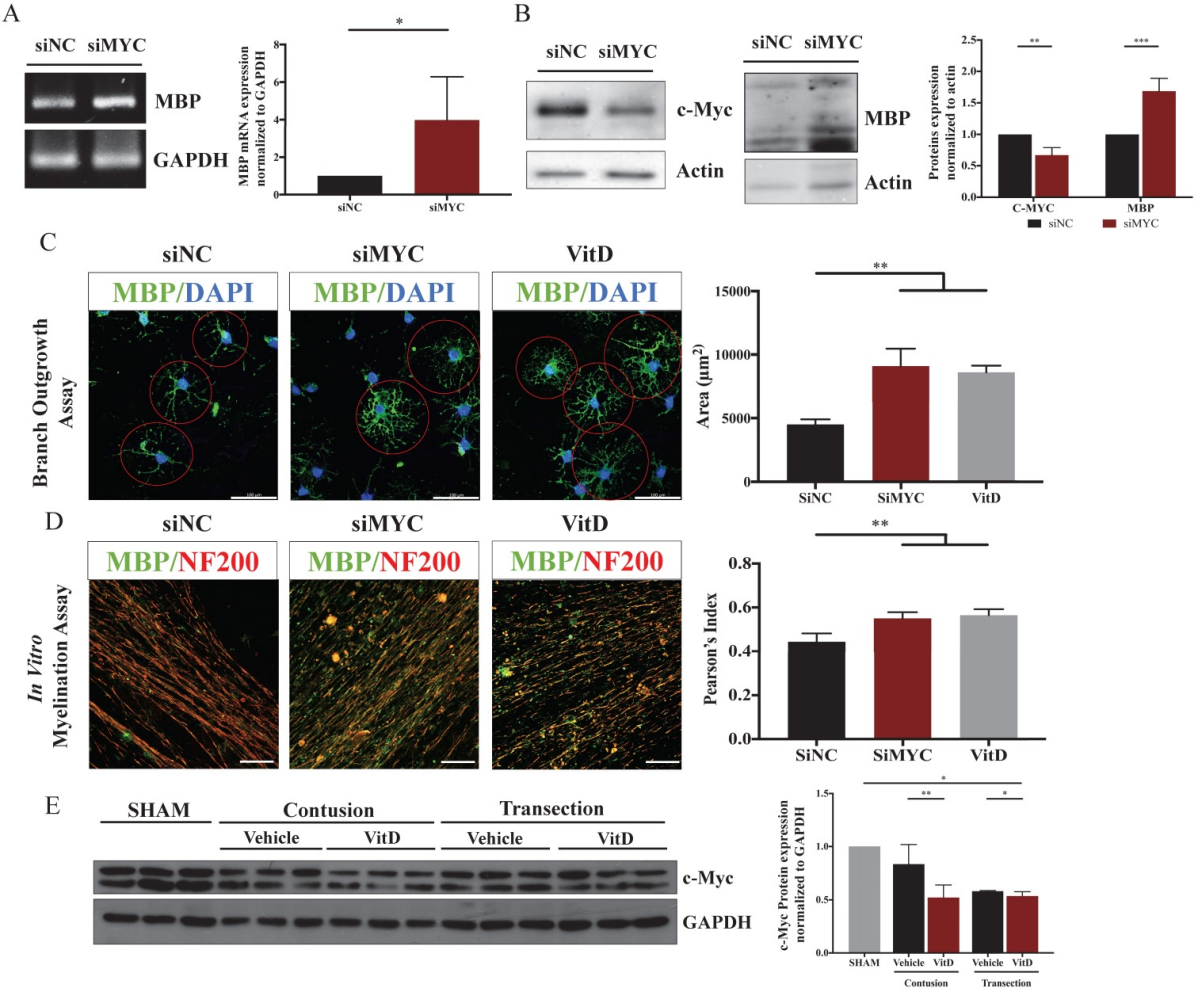
** c-Myc knockdown mimicked the VitD treatment and promoted OPCs differentiation and myelination *in vitro*. (A and B)** MBP mRNA and protein expression significantly increased when c-Myc was suppressed in OPCs. **(C)** Branch analysis showed that both c-Myc knockdown enhanced OPCs maturation, which mimicked the effect of VitD treatment (MBP: green; DAPI: blue; Scale bar = 100µm). **(D)** An *in vitro* myelination assay of a OPCs-DRG coculture demonstrated that c-Myc knockdown mimicked VitD treatment in promoting myelin sheath formation surrounding the DRG axons (MBP: green; NF200: red; Scale bar = 100µm). **(E)** c-Myc expression was reduced after VitD treatment in spinal cord specimens from rats with contusion and transection injury. Data are shown as mean ± SD.^ *^p < .05, ^**^p < .01, ^***^p < .001.

During VitD-stimulated OPCs differentiation, c-Myc protein expression was significantly suppressed compared to control (Figure 6A). As c-Myc is an important regulator in cell proliferation and differentiation, its role in OPCs were examined. To determine whether proliferation was suppressed, EdU and MTT assays were conducted. As expected, VitD treatment inhibited OPCs proliferation (Figure 6B,C). c-Myc was then knocked down to further investigate its impacts on OPCs proliferation and differentiation (Figure 6D). While c-Myc knockdown reduced OPCs proliferation (Figure 6E), MBP mRNA and protein expression levels were significantly elevated (Figure 7A,B). Morphological and *in vitro* myelination assays showed that c-Myc knockdown mimicked the effects of VitD treatment (Figure 7C,D). The *in vitro* results were further confirmed *in vivo* that c-Myc was suppressed in spinal cord specimens with VitD treatment after TSCI in a severity-dependent manner (Figure 7E). Taken together, it is plausible that c-Myc acts as a balance controller between OPCs proliferation and differentiation - suppressing c-Myc in OPCs may accelerate the differentiation of OLs and their *in vitro* myelination capabilities, at the expense of proliferation.

## Discussion

To our knowledge, this study is the first to demonstrate the therapeutic potential of VitD after TSCI by improving the integrity of myelin sheath through c-Myc suppression. In addition, it demonstrates the impact of pre-existing VitD-deficiency on functional and morphological recovery after TSCI. Although the mechanisms of injury and pathophysiological alterations differ between contusion and transection injury, VitD treatment effectively improved hindlimb movement in animals without prior VitD-deficiency irrespective of injury severity. However, cord-transected animals with prior VitD-deficiency did not seem to benefit from VitD supplementation. These animals only showed a marginal increase in serum VitD, which remained subnormal despite of VitD treatment when compared to contusion-injured animals. It is also interesting that significant improvement of motor recovery was only seen in animals whose serum VitD level had returned to normal level after TSCI, and in those who were no longer VitD-deficient (25(OH)D ≥30 nmol/L). This indicates that having sufficient VitD is essential for locomotor recovery which agrees with a previous study suggesting that VitD status could serve as an independent predictor of physical performance after TSCI 18. Whether animals with prior VitD-deficiency and severe injury would benefit from a higher dosage of VitD supplementation requires further investigations.

Previous studies have reported that the therapeutic effects of VitD treatment in neuronal injury 28 were mediated by various mechanisms, including immunomodulation, oxidative stress, and autophagy 14, 29. Pretreatment with calcitriol has also been shown to protect spinal cords from ischemia or reperfusion injury in rabbits 30. The role of VitD in promoting myelination has been well documented in peripheral nerve injury and multiple sclerosis models 31-34, but is less well defined in spinal cord injury. Recently, Gomez-Pinedo et al. showed that VitD promoted neural stem cell proliferation and differentiation into OL lineage cells in surgical lesions of the brain and increased MBP expression 35.

In the present study, significant demyelination was observed after contusion and transection injury the extent of which was associated with injury severity. Moreover, demyelination not only occurred at the injury site but also exhibited a bi-directionl damage extension to the rostral and caudal directions by at least 0.5cm from the epicenter. Surprisingly, there were no differences in myelin sheath thickness or MBP expression between VitD-depleted and normal rats although demyelination was more severe in VitD-deficient rats than in normal rats after TSCI, particularly in those with transection injury. Importantly, though, VitD treatment was able to enhance myelination in both normal and VitD-deficient animals with contusion injury whereas VitD-deficient rats with transection injury were less likely to benefit from VitD treatment. The overall findings were consistent with the functional outcome seen in rats, and that both motor improvement and remyelination only occur in post-TSCI animals with sufficient serum VitD (25(OH)D ≥30 nmol/L).

Apart from their role in myelin formation, OLs also provide trophic support to neurons and axons. Demyelination after TSCI often occurs due to apoptotic loss of OLs, leading to impaired saltatory neuronal signal transmission, neuronal death, and ultimately, functional deficits 3. To better understand the mechanism by which VitD protects myelin, we studied the effect of VitD in *in vitro* cultures of OPCs, OLs, and DRG. The results showed that VitD rescued OLs from apoptotic cell death. More importantly, VitD treatment promoted OPCs differentiation into OLs and further enhanced its myelination capability. In line with the findings reported by Gomez-Pinedo et al., these results suggest that remyelination through VitD supplementation could result from increased OL derivation from neural stem cells and increased differentiation from these OL progenitor cells 35.

Our study provides novel insights into VitD-induced regulation of cell differentiation at the molecular level. VitD and its analogues have been shown to enhance the differentiation of various cell types, including the differentiation of malignant leukemia cells into a more mature state with a macrophage-like phenotype 36, and the maturation of OLs upon activation of VitD signaling 25. The classical signaling pathway, where VitD binds to its nuclear receptor (VDR), has been shown to modulate cell differentiation by regulating its target genes 25, 37. Several non-genomic pathways that are independent of VDR, such as insulin growth factor, epidermal growth factor receptor, and Wnt/β-catenin signaling, are also known to be involved 38. In stem cells, c-Myc is considered an important regulator for maintaining stemness and self-renewal 39. We found that VitD promoted OPCs differentiation and inhibited OPCs proliferation by suppressing c-Myc expression in a manner like those reported in leukemia cells and myoblast where cell proliferation was inhibited during differentiation 40, 41. It has been suggested that the loss of c-Myc function in OPCs significantly enhances their morphological and functional maturation. It seems that c-Myc could be modulated by VitD through multiple mechanisms. VitD may act on the classical VDR pathway, which directly inhibits the transcriptional activation of c-Myc 42. A recent study also reported that following VDR activation, the transcription of MXD1 (which encodes for MAD, a potent antagonist of c-Myc) was highly upregulated 43. Therefore, VitD not only reduces the expression of c-Myc but also attenuates its transcriptional activity on downstream targets. Further mechanistic studies would be needed.

Taken together, our results show that VitD treatment is effective in promoting locomotor recovery after TSCI by preserving myelin integrity. A VitD-deficient cohort was also included in this study in view of the high prevalence of VitD deficiency in human 44. We found that while pre-existing VitD-deficiency did not impact on motor recovery in animals with mild TSCI, it can significantly reduce the therapeutic effect of VitD treatment following severe TSCI. To further determine the therapeutic effects of VitD in animals with severe injury, future investigations could consider pre-treatment with VitD to a supraphysiological level to determine whether pre-treatment could provide a protective effect from demyelination post-TSCI. While serum VitD level post-TSCI seem to be a critical determinant in motor recovery, it is not clear whether serum calcium was in fact involved in functional improvements. However, given that the serum VitD levels were only assessed at 8w post-TSCI, it is not clear whether the observed reduction of serum VitD occurs at an earlier stage or at the late stage during recovery.

From a clinical standpoint, this study highlights the importance of routine assessments of serum 25(OH)D levels in TSCI patients and provides a strong reason to test the effect of VitD treatment in the clinical setting as it is a safe, well-tolerated and readily accessible agent. The optimal treatment regimen may have to be guided by the individual patient's baseline serum VitD level, with the possibility that those with prior VitD-deficiency may require a higher dosage. The optimal serum VitD level, and any associated toxicity, would also need to be determined. Our study also demonstrates a novel function of VitD as a potent regulator of OLs survival and OPCs differentiation, and provided translational implications for combination therapy with OPCs transplantation and VitD supplementation to enhance the chances of OPCs survival while promoting *in situ* differentiation.

## Abbreviations

TSCI: traumatic spinal cord injury
VitD: vitamin D
OLs: oligodendrocytes
OPCs: oligodendrocyte precursor cells
DRG: dorsal root ganglion cells
MBP: myelin basic protein
